# The Influence of *Piper sarmentosum* Extract on Growth Performance, Intestinal Barrier Function, and Metabolism of Growing Chickens

**DOI:** 10.3390/ani13132108

**Published:** 2023-06-25

**Authors:** Luli Zhou, Yuhuan Lin, Ye Chang, Khaled Fouad Mohammed Abouelezz, Hanlin Zhou, Jian Wang, Guanyu Hou, Dingfa Wang

**Affiliations:** 1 Tropical Crops Genetic Resources Institute, Chinese Academy of Tropical Agricultural Sciences, Haikou 571101, China; zhoull@catas.cn; 2 Animal Health Supervision Institute of Hainan Province, Haikou 571100, China; yhlamcn@163.com; 3 College of Animal Science and Technology, Hainan University, Haikou 570228, China; changye970909@163.com (Y.C.); wangjian901@163.com (J.W.); 4 Department of Poultry Production, Faculty of Agriculture, Assiut University, Assiut 71526, Egypt; 5 Zhanjiang Experimental Station, Chinese Academy of Tropical Agricultural Sciences, Zhanjiang 524013, China; zhouhanlin8@163.com

**Keywords:** phyto-biotic extracts, feed additives, intestinal integrity, metabolism, growing chickens

## Abstract

**Simple Summary:**

This study assessed the effect of *Piper sarmentosum* extract addition on the growth performance, intestinal barrier function, and metabolism of growing chickens. It was found that dietary supplementation with *Piper sarmentosum* extract could enhance the anti-inflammatory capacity and intestinal mucosal barrier function of growing chickens.

**Abstract:**

In the poultry industry, there is an urgent need to evaluate and introduce natural, effective, and safe alternatives for synthetic antibiotics, which have been banned in most countries. The present study aimed to investigate the effects of dietary supplementation with *Piper sarmentosum* extract (PSE) on the growth performance, intestinal barrier function, and metabolism of growing chickens. A total of 400 seven-day-old female chicks were randomly assigned to four dietary treatments, each of which consisted of five replicates and twenty birds each. The four experimental treatments were fed a basal diet containing 0, 100, 200, and 300 mg PSE/kg (BC, PSE1, PSE2, and PSE3 groups), respectively. The experiment lasted for 28 days. The results showed that dietary supplementation with PSE had no significant effects on the final body weight, average daily gain (ADG), average daily feed intake (ADFI), and the ratio of ADFI to ADG (F/G) (*p* > 0.05). Compared with the BC group, dietary supplementation with 200–300 mg/kg PSE increased the villus height in the jejunum and ileum of chickens (*p* < 0.05). The PSE-treated groups significantly increased the mRNA expression of *Occludin*, *ZO-1*, and *Claudin-1* in the ileal mucosa of chickens (*p* < 0.05). In addition, a significant decrease in ileal TNF-α and IL-8 mRNA expression (*p* < 0.05) and a significant increase in IL-22 (*p* < 0.05) were observed in the PSE2 treatment compared to the BC group. Additionally, three gut metabolites (i.e., citrate, isocitrate, and spermine) showed significant differences among treatments (*p* < 0.05) and were involved in the tricarboxylic acid (TCA) cycle, the transfer of acetyl groups into mitochondria, and spermidine and spermine biosynthesis, respectively. In conclusion, the findings obtained here indicate that supplemental PSE can enhance the anti-inflammatory capacity and intestinal mucosal barrier function of chickens.

## 1. Introduction

The intestinal tract is the main organ enabling food digestion and absorption, and it also exerts an effective barrier function, preventing the invasion of pathogens [1]. Therefore, maintaining good intestinal functions is especially important for the growth and development of animals. In the post-antibiotic era, accumulative evidence has suggested that medicinal plant extracts, as potential replacements for antibiotics, can positively modulate gut health and improve animal growth performance, and these beneficial properties may be attributed to the presence of different bioactive compounds in plant extracts, such as alkaloids, phenolics, and other compounds [2,3,4,5,6].

*Piper sarmentosum* (PS) is a medicinal plant belonging to the Piperaceae family, which widely thrives within tropical and subtropical regions. It has been used in folk medicine to treat indigestion, toothache, hemorrhoids, malaria, and other diseases [7]. Some previous studies have revealed that *Piper sarmentosum* extracts (PSEs) have many biological functions such as antioxidant, analgesic, anti-microbial, and anti-inflammatory activities that might be related to various bioactive compounds in PSEs, such as alkaloids, amides, phenolics, and so on [8,9,10,11]. A recent study surveyed the chemical composition of PSE and listed more than 140 chemical compounds, including essential oils, alkaloids, flavonoids, lignans, steroids, and other compounds [12]. Additionally, previous research indicated that PSE supplementation in diets has been shown to effectively control coccidia infection in chicken broilers and improve poultry meat quality [13,14,15]. However, there are few studies on the effects of feeding PSE on the digestion, absorption, and metabolism of chickens. For its concentrated bioactive compounds, the PSE is believed to be a promising feed additive in poultry production. It is hypothesized that the dietary addition of a PSE is expected to display beneficial influences on growth performance, antioxidative status, metabolomics, and the health of the intestinal barrier of growing chickens. The present study aimed to explore the effect of PSE on growth, metabolomics, and the intestinal barrier function of chickens.

## 2. Materials and Methods

### 2.1. Obtaining PSE

A crude ethanol-extracted solution of PS and its ingredients were obtained from our previous study [11]. The extracted solution was then concentrated by evaporation (RE-3000A, Shanghai Yarong Biochemistry Instrument Factory, Shanghai, China) to obtain the PSE. Subsequently, the resulting PSE was blended with silica until a homogeneous mix of powder was obtained (PSE:silica = 1:1).

### 2.2. Experimental Design

A total of 400 seven-day-old female Danzhou chicks (average initial body weight: 51.38 ± 2.25 g) were randomly allocated to 20 floor pens (with an equal space: 1.2 m × 1.2 m). The design of the experiment was a single-factor completely randomized design, which included four treatments with five replication pens per treatment (twenty chicks per pen). The control group (BC) was fed the basal diet, while the other three groups were fed the same basal diet supplemented with PSE at 100 (PSE1), 200 (PSE2), or 300 mg/kg (PSE3), respectively. The composition of the basal diet and associated nutrient levels are presented in Table 1. Chickens were offered ad libitum access to feed and water. The experimental period lasted for 28 days. This research on live animals met the guidelines of and was approved by the Institutional Animal Care and Use Committee of the Chinese Academy of Tropical Agricultural Sciences (approval number CATAS-20220301-1).

### 2.3. Sample Collection

At the end of the experiment, one chicken per replicate was randomly selected, and blood samples were collected from the jugular vein in non-anticoagulation tubes. The collected blood was centrifuged in a benchtop centrifuge at 4000× *g* for 10 min to obtain the sera and then stored at −80 °C until analysis. The chickens were immediately euthanized by cervical dislocation. About 2 cm length of the jejunum and ileum were collected, rinsed in 0.1 M phosphate-buffered saline, and then fixed in 10% formaldehyde-phosphate buffer for histological analysis. The ileal mucosa samples were scraped into a 2 mL tube, snap-frozen in liquid nitrogen, and stored at −80 °C for subsequent total RNA isolation. The contents of the jejunum and ileum were immediately collected and mixed with the intestinal contents, and the collected samples were snap-frozen in liquid nitrogen and stored at −80 °C.

### 2.4. Intestinal Morphology Analysis

The fixed tissue samples were processed by an ascending series of ethanol, cleared with xylene, and then infiltrated with paraffin wax. Histological sections (5 μm thickness) were stained with hematoxylin and eosin (HE) for histological evaluation under light microscopy (100×, Olympus, ON, Canada). A total of 10 well-oriented intact villus height and crypt depth measurements were selected and measured for each intestinal cross-section, and the means of these measurements were calculated for each sample.

### 2.5. Biochemical Parameters Analysis

The serum levels of endotoxin 1 (ET-1), diamine oxidase (DAO), D-lactic acid (D-LA), TNF-α, and IL-1β, -8, and -22 were determined by enzyme-linked immunosorbent assay (ELISA) kits, according to the manufacturer’s instructions (Jiancheng Bioengineering Institute, Nanjing, China).

### 2.6. RNA Extraction and Determination of mRNA Expression by RT-PCR

Total RNA was extracted from ileal mucosa samples using a total RNA isolation kit according to the recommendations of the manufacturer (Vazyme, Nanjing, China). The quality and quantity of the RNA were assessed by an Agilent Bioanalyzer (Agilent Technologies, Shanghai, China). cDNA was produced from 1 μg of total RNA in each sample using a cDNA synthesis kit (Vazyme, Nanjing, China). Real-time PCR was performed by an ABI 7300 real-time PCR system (Applied Biosystems) using a ChamQ Universal SYBR qPCR Master Mix (Vazyme, Nanjing, China) with a total volume of 10 μL to analyze the mRNA expression of Occludin, Claudin-1, zonula occludens-1 (ZO-1), TNF-α, and IL-1β, -8, and -22 in the ileum mucosa of chickens. Data normalization was accomplished using glyceraldehyde 3-phosphate dehydrogenase (GAPDH) as a housekeeping gene. Primers were commercially synthesized by Sangon Biotech (Shanghai, China), and sequences were shown as follows: Occludin (NM_205128.1), F: AGACGCGCAGTAAGATCTGG, R: CACGTTCTTCACCCACTCCT, 104 bp; Claudin 1 (NM_001013611.2), F: ACCCGTTAACACCAGATTTGAG, R: TGGGTAGGATGTTTCACTCCG, 124 bp; ZO-1 (NM_015278981.2), F: TCTGAACCCGTTAGGGAGGAT, R: CTGTATACCGGCTGAGAAGCA, 143 bp; IL-1β(XM_015297469.1), F: CTTCCTTCCAGCGCTCCTT, R: CCGTAGAAGGTCTCTTCGCT, 189 bp; TNF-α (NM_204267), F: TTCCTGCTGGGGTGCATAG, R: AAGAACCAACGTGGGCATTG, 106 bp; IL-8 (HM_179639.1), F: GCAAGGTAGGACGCTGGTAA, R: TTGGCGTCAGCTTCACATCT, 110 bp; IL-22 (NM_001199614.1), F: ACGTCAACATCAGGGAGAACAA, R: CACATCCTCAGCATACGGGT, 116 bp; GAPDH (NM_204305.1), F: ACTGTCAAGGCTGAGAACGG, and R: ACCTGCATCTGCCCATTTGA, 151 bp. The specificity of the PCR product was assessed by melting curve analysis. All reactions were run in triplicate. The 2^−ΔΔCT^ method was used to analyze the relative expression of genes compared with the blank control [16].

### 2.7. Untargeted Metabolomic Analysis

The metabolomic analyses were performed on the serum and intestinal contents, respectively. The details about these sample processing, detection methods, and data analyses have been described in our previously published study [17].

For serum, 100 μL of each sample was mixed with 400 μL of methanol (TEDIA, Fairfield, OH, USA) and then vortexed for 2 min. For intestinal contents, 500 μL of methanol was added to each sample (100 mg) and mixed for 5 min in a vortex. The obtained homogenates were centrifuged at 20,000× *g* for 10 min (4 °C). Subsequently, 400 μL of metabolite-containing supernatant was transferred into an Eppendorf tube and dried in a vacuum concentrator (Speed-Vac), and then re-dissolved in 100 μL of acetonitrile (MERCK, Darmstadt, Germany) for metabolic analysis.

Briefly, instrumental analysis was performed on an LC-30AD series ultra-high-performance liquid chromatography (UHPLC) system (NexeraTM, Shimadzu, Kyoto, Japan) coupled to a hybrid quadrupole time-of-flight mass spectrometer (QTOF-MS) (TripleTOF5600, Sciex, Concord, ON, USA) interface with a Turbo VTM electrospray ionization (ESI) source that operated in positive and negative modes, respectively. Chromatographic separation was carried out on an XBridge HILIC (2.1 × 100 mm, 3.5 μm) column (Waters, Milford, MA, USA) maintained at 30 °C. The mobile phases consisted of 50% acetonitrile in 0.1% formic acid (A) (SIGMA, Deisenhofen, Germany) and 95% acetonitrile in 0.1% formic acid (B). Samples were eluted at a flow rate of 250 μL/min with a gradient elution of the mobile phase starting at 80% B and decreased to 20% B in 24 min, increased to 80% B at 24.5 min, and held for 8.5 min, for a total run of 33 min. The source parameters were drying gas temperature, 550 °C; spray voltage, 5000 V; and atomization gas, auxiliary heating gas, and curtain gas pressure, 45 psi, 45 psi, and 35 psi, respectively. Based on the original acquisition files, a pre-processing step with MarkerView software (Sciex, USA) was performed for automated baseline correction and alignment of all extracted mass peaks across all samples.

### 2.8. Statistical Analysis

The replicate was considered as the experimental unit. Statistical analyses of results were evaluated by using the one-way analysis of variance (ANOVA), which was performed using SPSS 23.0 (IBM-SPSS Inc., Chicago, IL, USA). The results were presented as means ± standard deviation (SD). Significant differences were evaluated by Tukey’s multiple comparisons test at *p* < 0.05.

The metabolomic data among multiple groups were used for principal component analysis (PCA), supervised partial least-squares discriminant analysis (PLS–DA), metabolic enrichment analysis, and one-way ANOVA with Fisher’s LSD post hoc analysis by MetaboAnalyst 5.0 software (http://www.metaboanalyst.ca (accessed on 25 May 2022)), where *p* < 0.05 was considered significant.

## 3. Results

### 3.1. Growth Performance

The growth performance results of the chickens that were fed graded PSE levels between 7 and 35 days of age are shown in Table 2. During the whole experimental period, dietary supplementation with graded PSE levels had no significant effect on the final body weight, average daily gain (ADG), average daily feed intake (ADFI), and the ratio of ADFI to ADG (F/G) (*p* > 0.05).

### 3.2. Intestinal Morphology

The effect of PSE on the intestinal morphology of broilers is presented in Table 3. Compared with the BC group, dietary supplementation with PSE at 200 mg/kg increased the villus height and ratio of the villus height to crypt depth (V/C) in the jejunum of chickens (*p* < 0.05). Meanwhile, dietary supplementation with 200–300 mg/kg of PSE increased the villus height in the ileum of chickens (*p* < 0.05).

### 3.3. Mucosal Barrier Function

The mucosal barrier function of chickens was reflected by serum levels of DAO, D-LA, and ET-1, and the mRNA expression of *Occludin*, *ZO-1*, and *Claudin-1* in the ileal mucosa. As shown in Table 4, dietary supplementation with PSE had no significant effect on the levels of DAO, D-LA, and ET-1 in the serum of chickens (*p* > 0.05). However, dietary supplementation with PSE significantly increased the mRNA expression of *Occludin*, *ZO-1*, and *Claudin-1* in the ileum mucosa of chickens (*p* < 0.05).

### 3.4. Anti-Inflammation Effect

As displayed in Table 5, although there were no significant differences (*p* > 0.05) of cytokine concentrations in serum among the treatments, there were trends toward higher serum levels of IL-22 and lower TNF-α, IL-8, and IL-1β in the PSE-treated groups compared with the BC group. Similarly, the administration of PSE reduced ileal TNF-α, IL-8, and IL-1β mRNA expressions and increased IL-22 expression in comparison to the BC group. Notably, we observed a significant decrease in ileal TNF-α and IL-8 gene expression levels (*p* < 0.05) and a significant increase in the gene expression level of IL-22 (*p* < 0.05) in the PSE2 group compared to the BC group. Altogether, these results showed that the three PSE-treated groups exerted an effective anti-inflammation effect and that adding 200 mg/kg of PSE to the diets was the most effective.

### 3.5. Untargeted Metabolomics Analysis

**Figure 1:**
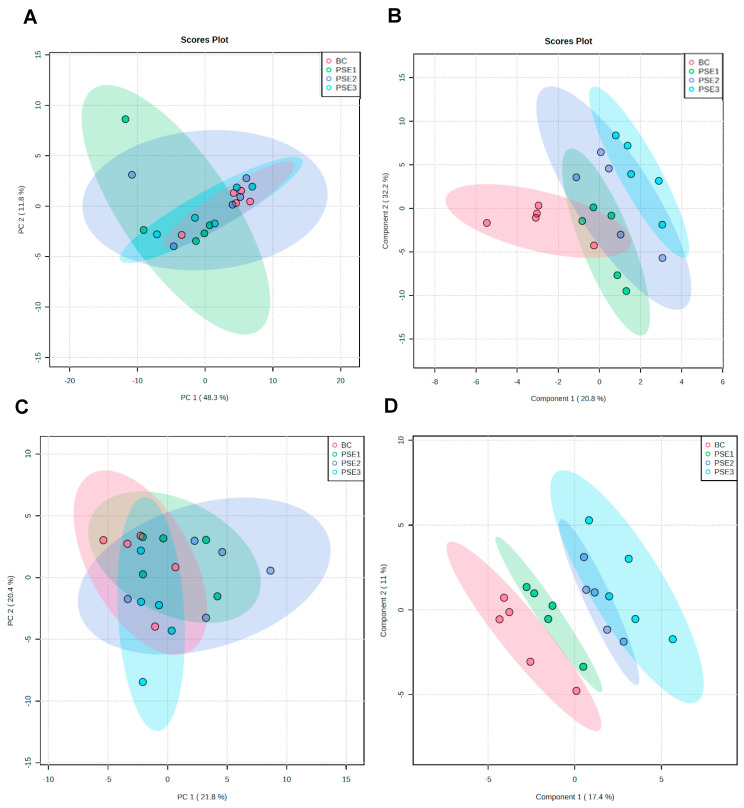
Multivariate statistical analysis of the UHPLC–QTOF/MS–based metabolite data among these four treatments. (**A**) PCA score plot for all serum samples; (**B**) PLS–DA score plot for all serum samples; (**C**) PCA score plot for all intestinal content samples; and (**D**) PLS–DA score plot for all intestinal content samples.

PCA and PLS–DA were initially carried out to visualize the overall alterations of all serum and gut metabolites in the different groups, respectively (Figure 1). The PCA score plot shows principal components 1 and 2 (PC1 and PC2). As described in Figure 1A,C, the unsupervised PCA scores plot based on serum and gut metabolites explained almost 60.1% (48.3% PC1 and 11.8% PC2) and 42.2% (21.8% PC1 and 20.4% PC2) of data variability, respectively. Further, supervised PLS-DA observed that there was a partial overlap of the levels of serum metabolites between groups (Figure 1B). However, there were evidently different trends in the levels of the gut metabolites among groups, which reflected the remarkably distinct metabolic status, with the exception of the PSE2 group versus the PSE3 group (Figure 1D).

**Figure 2:**
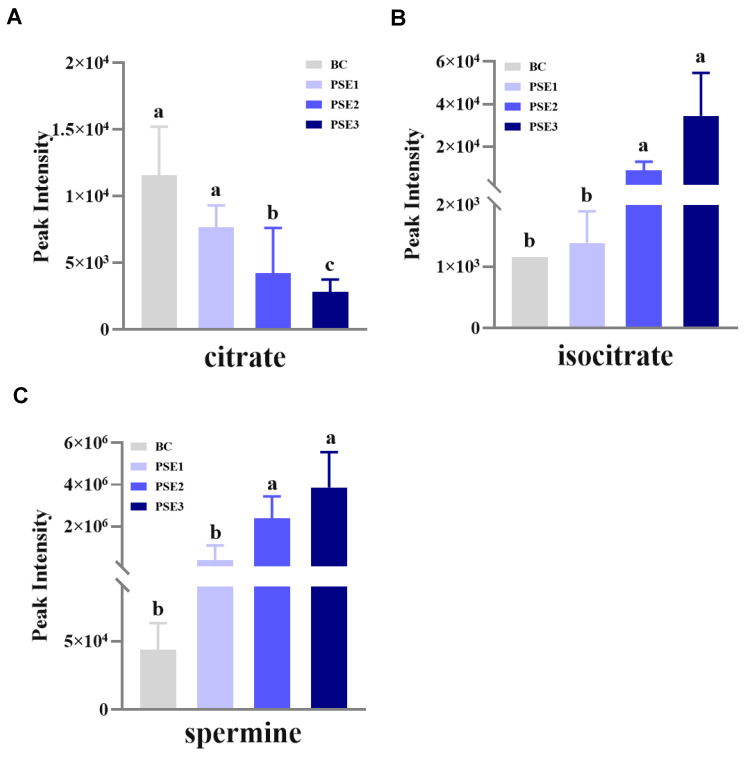
Histogram analysis of the detected differential metabolites in the intestinal content of growing chickens at 35 d of age from each treatment. Results are means ± standard deviation of the mean (*n* = 5). BC: chickens fed with a basal diet; PSE1, PSE2, PSE3: chickens fed with the same basal diet supplemented with 100, 200, and 300 mg/kg of PSE, respectively. (**A**) Peak intensity of citrate in the metabolomics analysis; (**B**) Peak intensity of isocitrate in the metabolomics analysis; (**C**) Peak intensity of spermine in the metabolomics analysis; different superscript lowercase letters in each bar represent significant differences.

**Figure 3:**
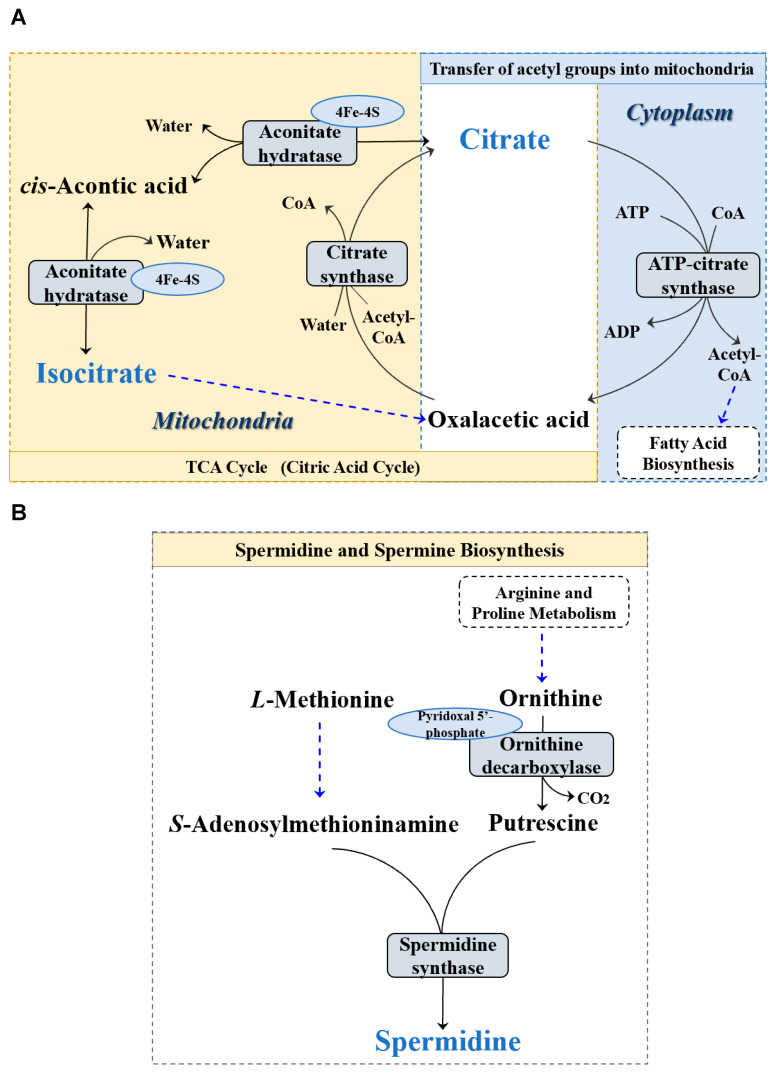
Pathway enrichment analysis of the differential metabolites of the intestinal contents in the four treatments. (**A**) Pathway of TCA cycle; (**B**) Pathway of spermidine and spermine biosynthesis.

We then performed metabolomic analyses on serum and gut metabolites among the groups, respectively. It was found only three gut metabolites (i.e., citrate, isocitrate, and spermine) were significantly different among the groups (*p* < 0.05) (Figure 2). Interestingly, the results showed that the PSE2 and PSE3 groups significantly downregulated the level of citrate (*p* < 0.05) (Figure 2A) and upregulated isocitrate (*p* < 0.05) (Figure 2B) and spermine (*p* < 0.05) (Figure 2C) compared with the BC and PSE1 groups. Meanwhile, as the addition of PSE in diets increased, the upregulation or downregulation of the three differential gut metabolites occurred in a dose-dependent manner. Further, based on the SMPD pathway database (https://smpdb.ca/ (accessed on 25 May 2022)), pathway analysis was performed to predict the significantly enriched pathways involving the three differential metabolites. As shown in Figure 3, the analysis revealed that citrate and isocitrate had the highest correlation with the tricarboxylic acid (TCA) cycle. Additionally, it was also noted that citrate and spermine were involved in the transfer of acetyl groups into mitochondria and spermidine and spermine biosynthesis, respectively.

## 4. Discussion

Some studies revealed that phenolics possess affinities to nutrient transporters, such as PEPT1 and SGLT1, and upregulated their gene expression levels, which in turn enhanced the animals’ body weight gain [18,19]. Our previous research also demonstrated that dietary supplementation with 50 mg/kg of PSE increased the ADG of weaned piglets [10]. In the present study, the growth performance of chickens was not affected significantly by PSE supplementation. Our 28-day experimental period (which began with seven-day-old chicks) was maybe not sufficient to display a significant influence on growth performance due to the slow growth rate of Danzhou chickens. Nevertheless, the final body weight and ADG in the PSE2 and PSE3 groups have shown an increasing trend.

The intestinal morphological parameters including villus height, crypt depth, and the ratio of villus height to crypt depth were commonly used as important indicators for evaluating gut health status [20]. Our findings showed that diets supplemented with PSE increased the villus height of the jejunum and ileum in chickens, which may have contributed to enhancing intestinal absorptive capacity [21,22]. Furthermore, a high ratio of villus height to crypt depth implies better intestinal digestive and absorption functions [23]. Of these, only the PSE2 group significantly increased the ratio of villus height to crypt depth compared with the other three groups; this implies that dietary supplementation with PSE at 200 mg/kg appears to be an optimal dosage for improved gut health of chickens.

It is well known that multiple tight junction proteins, such as Claudin-1, Occludin, and ZO-1, are essential components of the physical barrier responsible for maintaining intestinal integrity [24,25,26]. Many proinflammatory cytokines, such as TNF-α and IL-1β, -6, -8, and -18, are important in cell-mediated immune response activity and are usually secreted during the early phase of acute and chronic inflammatory diseases, whereas IL-22 is an anti-inflammatory cytokine that targets epithelial cells and may play an immunological role as a barrier against pathogens [27,28]. The results obtained in this study revealed that the addition of PSE in the diets of the experimental chickens can fortify intestinal barrier function by increasing the mRNA expression of *Claudin-1*, *Occludin*, and *ZO-1* in the ileum. In addition, we also found that compared with the BC group, dietary supplementation with 100–300 mg/kg of PSE effectively decreased the serum and ileal mucosal mRNA levels of TNF-α, IL-1β, and IL-8 while resulting in an increase in IL-22. As reported, PSE is a complex mixture, and its primary active constituents include phenolics and alkaloids, which possess various biological effects such as anti-inflammatory, antioxidative, and anti-microbial functions [11,29]. We hypothesized that multiple active components in PSE may cooperatively inhibit the growth of harmful bacteria in the intestine of chickens, thus providing a suitable environment and improving intestinal development and barrier function.

Metabolically, it was found in this study that the addition of PSE to the diets tended to downregulate the citrate level and upregulate the isocitrate and spermine levels in the intestinal content of chickens. This trend became even more remarkable when diets were supplemented with PSE at or above 200 mg/kg. Some studies have confirmed that citrate can be transported from the mitochondria and then converted back into acetyl-CoA for fatty acid synthesis in the transfer of acetyl groups into the mitochondrial pathway. On the other hand, the citrate is also converted to cis-aconitic acid and then isocitrate via the enzyme aconitase in the TCA cycle. Our results indicated that PSE seems to increase the activity of the enzyme aconitase hydratase with concurrent activation of the TCA cycle, which is conducive to providing adequate energy for enterocyte proliferation and intestinal development, as well as reducing oxidative stress in the gut [30,31]. Moreover, the high level of spermine in the intestine of chickens in the PSE-treated groups could also be beneficial for protection from oxidative damage and maintenance of enterocyte membrane structure and function [32].

## 5. Conclusions

Altogether, the results indicated that dietary supplementation with PSE (particularly at 200 mg/kg) enhanced the anti-inflammatory capacity and intestinal mucosal barrier function during the brooding phase of Danzhou chickens.

## Figures and Tables

**Table 1:** Ingredient and nutrient levels of the basal diet of growing chickens (%, air-dry basis).

Items	Contents
Ingredients	
Corn	60.00
Soybean meal	35.50
Limestone (Ca% ≥ 35.8%)	1.70
Calcium hydrogen phosphate	1.30
Premix ^1^	1.50
Total	100.00
Nutrient levels ^2^	
Metabolic energy (MJ/kg)	12.01
Crude protein	21.00
Lysine	1.11
Methionine	0.53
Calcium	1.05
Total phosphorus	0.58

^1^ Premix supplied the following per kilogram of diet: vitamin A, 11,000 IU; vitamin E, 20 IU; vitamin K, 3 mg; vitamin D, 3000 IU; vitamin B1, 2 mg; vitamin B2, 8 mg; vitamin B12, 0.04 mg; Fe, 65 mg; Cu, 10 mg; Mn, 77 mg; Zn, 70 mg; pantothenic acid, 19 mg; folic acid, 1.1 mg. ^2^ The nutrient levels are calculated values. The values of lysine, methionine, and calcium mean the total content in the diet.

**Table 2:** The effect of PSE on the growth performance of growing chickens.

Item	Groups				*p*-Value
	BC	PSE1	PSE2	PSE3	
Initial body weight, g	51.90 ± 3.02	50.73 ± 1.70	50.30 ± 1.53	50.00 ± 0.45	0.438
Final body weight, g	264.72 ± 15.99	262.83 ± 16.19	285.40 ± 16.77	276.42 ± 1.30	0.074
Average daily gain (ADG), g	7.60 ± 0.55	7.57 ± 0.62	8.40 ± 0.55	8.09 ± 0.06	0.050
Average daily feed intake (ADFI), g	23.60 ± 1.62	22.11 ± 0.42	24.22 ± 2.30	24.59 ± 0.57	0.071
ADFI/ADG (F/G)	3.12 ± 0.35	2.94 ± 0.25	2.88 ± 0.12	3.04 ± 0.06	0.365

BC: chickens fed with a basal diet; PSE1, PSE2, PSE3: chickens fed with the same basal diet supplemented with 100, 200, and 300 mg/kg of PSE, respectively.

**Table 3:** The effect of PSE on the mucosal structure of the jejunum and ileum of growing chickens.

Item	Groups				*p*-Value
	BC	PSE1	PSE2	PSE3	
Jejunum					
Villus height, μm	576.91 ^b^ ± 112.61	855.06 ^a^ ± 43.56	909.35 ^a^ ± 139.75	891.06 ^a^ ± 15.44	<0.001
Crypt depth, μm	147.63 ^b^ ± 8.34	206.76 ^a^ ± 39.74	149.24 ^b^ ± 15.57	220.38 ^a^ ± 7.57	<0.001
Villus height/Crypt depth	3.92 ^b^ ± 0.80	4.36 ^b^ ± 0.71	6.18 ^a^ ± 1.06	4.05 ^b^ ± 0.07	<0.001
Ileum					
Villus height, μm	587.62 ^b^ ± 29.58	666.92 ^ab^ ± 116.87	751.97 ^a^ ± 115.77	753.03 ^a^ ± 61.77	0.006
Crypt depth, μm	134.17 ± 28.62	141.51 ± 29.55	159.05 ± 27.50	172.00 ± 30.05	0.157
Villus height/Crypt depth	4.49 ± 0.63	4.82 ± 0.92	4.96 ± 1.48	4.50 ± 0.84	0.819

^a,b ^Mean values within a row with no common superscripts differ significantly at *p* < 0.05. *n* = 5. BC: chickens fed with a basal diet; PSE1, PSE2, PSE3: chickens fed with the same basal diet supplemented with 100, 200, and 300 mg/kg of PSE, respectively.

**Table 4:** The effect of PSE on the intestinal permeability of growing chickens.

Item	Groups				*p*-Value
	BC	PSE1	PSE2	PSE3	
Serum D-LA, ET-1 concentrations, and DAO activity					
D-LA, pg/mL	30.07 ± 2.82	23.73 ± 3.20	27.78 ± 6.05	30.21 ± 4.05	0.091
ET-1, ng/L	190.03 ± 6.55	184.09 ± 23.64	197.97 ± 4.67	181.76 ± 9.55	0.262
DAO, pg/mL	23.84 ± 1.62	23.11 ± 1.60	22.37 ± 0.98	23.00 ± 1.05	0.415
mRNA expression of ileal tight junction proteins					
*Occludin*	1.00 ^b^ ± 0.00	1.46 ^a^ ± 0.30	1.11 ^ab^ ± 0.24	1.35 ^ab^ ± 0.32	0.039
*ZO-1*	1.00 ^b^ ± 0.00	1.38 ^a^ ± 0.24	1.47 ^a^ ± 0.11	1.60 ^a^ ± 0.13	<0.001
*Claudin-1*	1.00 ^b^ ± 0.00	1.12 ^ab^ ± 0.11	1.41 ^a^ ± 0.26	1.23 ^ab^ ± 0.22	0.015

^a,b ^Mean values within a row with no common superscripts differ significantly at *p* < 0.05. *n* = 5. D-LA, D-lactic acid; ET-1, endotoxin 1; DAO, diamine oxidase; *ZO-1*, zonula occludens-1. BC: chickens fed with a basal diet; PSE1, PSE2, PSE3: chickens fed with the same basal diet supplemented with 100, 200, and 300 mg/kg of PSE, respectively.

**Table 5:** The effect of different dietary levels of PSE on the cytokine profiles of growing chickens.

Item	Groups				*p*-Value
	BC	PSE1	PSE2	PSE3	
Cytokine concentration in serum, pg/mL					
TNF-α	63.94 ± 6.86	61.05 ± 11.86	59.32 ± 4.83	63.00 ± 9.23	0.835
IL-1β	529.82 ± 94.84	515.83 ± 57.36	466.09 ± 67.31	511.07 ± 97.27	0.637
IL-8	93.36 ± 21.63	89.35 ± 8.67	68.10 ± 9.05	78.33 ± 13.84	0.053
IL-22	299.63 ± 50.67	316.15 ± 27.92	346.21 ± 51.58	323.14 ± 14.71	0.342
mRNA expression of cytokines in the ileum					
TNF-α	1.00 ^a^ ± 0.00	0.90 ^ab^ ± 0.13	0.66 ^b^ ± 0.24	0.88 ^ab^ ± 0.22	0.045
IL-1β	1.00 ± 0.00	0.79 ± 0.20	0.75 ± 0.15	0.86 ± 0.13	0.064
IL-8	1.00 ^a^ ± 0.00	0.78 ^ab^ ± 0.15	0.73 ^b^ ± 0.04	0.96 ^ab^ ± 0.25	0.025
IL-22	1.00 ^b^ ± 0.00	1.10 ^ab^ ± 0.21	1.46 ^a^ ± 0.33	1.27 ^ab^ ± 0.17	0.018

^a,b^ Mean values within a row with no common superscripts differ significantly at *p* < 0.05. *n* = 5. BC: chickens fed with a basal diet; PSE1, PSE2, PSE3: chickens fed with the same basal diet supplemented with 100, 200, and 300 mg/kg of PSE, respectively.

## Data Availability

The data were not deposited in an official repository. The data that support the findings are available from the corresponding authors upon request.
